# Cancer stem-like sphere cells induced from de-differentiated hepatocellular carcinoma-derived cell lines possess the resistance to anti-cancer drugs

**DOI:** 10.1186/1471-2407-14-722

**Published:** 2014-09-27

**Authors:** Noriaki Hashimoto, Ryouichi Tsunedomi, Kiyoshi Yoshimura, Yusaku Watanabe, Shoichi Hazama, Masaaki Oka

**Affiliations:** Department of Digestive Surgery and Surgical Oncology, Yamaguchi University Graduate School of Medicine, 1-1-1 Minami-Kogushi, Ube, Yamaguchi, 755-8505 Japan

**Keywords:** Cancer stem cell, HCC, Sphere, Chemoresistance

## Abstract

**Background:**

Cancer stem cells (CSCs) are thought to play important roles in therapy-resistance. In this study, we induced cancer stem-like cells from hepatocellular carcinoma (HCC) cell lines using a unique medium, and examined their potential for resistance to anti-cancer drugs.

**Methods:**

The human HCC cell lines SK-HEP-1 (SK), HLE, Hep 3B, and HuH-7 were used to induce cancer stem-like cells with our sphere induction medium supplemented with neural survival factor-1. *NANOG* and *LIN28A* were examined as stemness markers. Several surface markers for CSC such as CD24, CD44, CD44 variant, and CD90 were analyzed by flow-cytometry. To assess the resistance to anti-cancer drugs, the MTS assay, cell cycle analysis, and reactive oxygen species (ROS) activity assay were performed.

**Results:**

Poorly differentiated HCC derived SK and undifferentiated HCC derived HLE cell lines efficiently formed spheres of cells (SK-sphere and HLE-sphere), but well-differentiated HCC-derived HuH-7 and Hep 3B cells did not. SK-spheres showed increased *NANOG*, *LIN28A*, and *ALDH1A1* mRNA levels compared to parental cells. We observed more CD44 variant-positive cells in SK-spheres than in parental cells. The cell viability of SK-spheres was significantly higher than that of SK cells in the presence of several anti-cancer drugs except sorafenib (1.7- to 7.3-fold, each *P* < 0.05). The cell cycle of SK-spheres was arrested at the G0/G1 phase compared to SK cells. SK-spheres showed higher *ABCG2* and *HIF1A* mRNA expression and lower ROS production compared to parental cells.

**Conclusion:**

Our novel method successfully induced cancer stem-like cells, which possessed chemoresistance that was related to the cell cycle, drug efflux, and ROS.

**Electronic supplementary material:**

The online version of this article (doi:10.1186/1471-2407-14-722) contains supplementary material, which is available to authorized users.

## Background

Hepatocellular carcinoma (HCC) is the sixth most common and the third most deadly cancer worldwide affecting 1 million individuals annually [1]. Most potentially curative therapies for HCC such as surgical resection, transplantation and ablation therapy are of limited efficacy in advanced stages, and the recurrence rate after these treatments is 40 to 80% within 5 years [2, 3]. Various types of post-operative therapies, including transarterial lipiodol chemoembolization, systemic or focal chemotherapy, α-interferon, adoptive immunotherapy, and oral acyclic retinoid acid have been used in HCC patients following curative treatment. However, the benefits of adjuvant therapy have not been definitively demonstrated. Therefore, new adequate adjuvant therapy is needed to improve survival.

Recent research has focused on cancer stem cells (CSCs) to increase our understanding of the characteristics of cancers including HCC. CSCs possess stem cell properties such as a self-renewal capacity, tumor-initiating ability, higher tumorigenicity, metastatic potential, and chemoresistance [4]. To investigate CSC properties such as tumor initiation and recurrence, many studies have examined methods for isolation of CSCs from cancer specimens or cell lines. As a result, a side-population of cells and several proteins as markers for CSCs have been identified [5, 6]. These CSC markers include CD44, CD133, CD90, CD13, aldehyde dehydrogenase (ALDH), oval cell marker OV6, and epithelial cell adhesion molecule [7–14]. However, resulting yields of CSCs have been too low for further analysis [15]. Alternatively, as a functional approach, enrichment of a potential CSC subpopulation using sphere formation with a conditioned serum-free culture system supplemented with epidermal growth factor (EGF) and basic fibroblast growth factor (bFGF) was considered useful for enriching CSCs. Sphere cells possess the capacity for self-renewal and tumorigenicity and are thus considered to be CSCs [16, 17].

Initially, CSCs and their progenitor cancer cells were thought to form a unidirectional hierarchy, with CSCs located at the top of the hierarchy [18]. Consistent with the hierarchy model, isolation of CSCs has mainly been performed with well-differentiated HCC cell lines, which are thought to include relatively large quantity of CSCs compared to cell lines derived from advanced HCC [17, 19–22]. On the other hand, recent breast cancer studies have revealed the plasticity of cancer cells in which differentiated cancer cells can transform to attain cancer stem-like properties via epithelial-mesenchymal transition (EMT) [23]. Moreover, higher grade glioma or oral squamous cell carcinoma cells can form a sphere of cells with higher CSC marker expression compared to lower grade cancers [24, 25]. Regarding HCC, previous studies have focused on the isolation of CSCs from less advanced HCC. In this study, based on the concept of cancer plasticity, we tried to induce CSCs from cell lines derived from advanced HCC using a unique sphere induction medium.

## Methods

### Cell lines

The human HCC cell lines SK-HEP-1 (SK), HLE, HuH-7, and Hep 3B were purchased from the Health Science Research Resources Bank (Osaka, Japan) and American Type Culture Collection (Rockville, MD). The SK and HLE cell lines are poorly differentiated and undifferentiated HCC derivatives, respectively. The HuH-7 and Hep 3B cell lines are well-differentiated HCC derivatives. Cells were cultured in DMEM (Nissui Pharmaceutical, Tokyo, Japan) with 10% heat-inactivated fetal bovine serum (FBS, Life Technologies, Tokyo, Japan) supplemented with penicillin (100 U/mL), streptomycin (100 μg/mL), and sodium bicarbonate (1.5 g/L) at 37°C in a humidified atmosphere of 5% CO_2_ in air.

### Induction of sphere cells

Cells were suspended in the sphere induction medium, which is based on neural stem cell medium. The basal medium for the sphere induction medium is DMEM/F12 (Sigma-Aldrich, Tokyo, Japan) supplemented with 10 mM HEPES (Sigma-Aldrich), 1× antibiotic-antimycotic solution (Sigma-Aldrich), 0.6% glucose (Sigma-Aldrich), 1 mg/mL transferrin, 250 μg/mL insulin (Sigma-Aldrich), 0.6 mM putrescine (Sigma-Aldrich), 0.3 μM sodium selenite (Sigma-Aldrich), and 0.2 μM progesterone (Sigma-Aldrich). Complete sphere induction medium was prepared by adding 2 μg/mL heparin (Sigma-Aldrich), 10 ng/mL human recombinant EGF (Sigma-Aldrich), 10 ng/mL bFGF (Merck Millipore, Tokyo, Japan), 10 ng/mL leukemia inhibitory factor (LIF, Merck Millipore), 60 μg/mL *N*-acetyl-L-cysteine (NAC, Sigma-Aldrich), and 1/50 vol. neural survival factor-1 (NSF-1, Lonza, Tokyo, Japan) to the basal medium. Briefly, cells were collected and washed to remove serum and then cultured in the sphere induction medium at 37°C in a humidified atmosphere of 5% CO_2_ in air. The next day, induction was begun, and floating cells were transferred into a hydrophilic ultra low attachment flask (Corning, Corning, NY).

### Semi-quantitative real-time RT-PCR

Semi-quantitative real-time PCR (semi-qRT-PCR) was performed as described previously with minor modifications [26]. RT-PCR amplification was performed using LightCycler 480 Probe Master (Roche Diagnostics, Tokyo, Japan) and Universal ProbeLibrary Probes (Roche Diagnostics) in a LightCycler System Version 3 (Roche Diagnostics). Primers and probes are listed in Additional file 1: Table S1. Amplification was performed according to a two-step cycle procedure consisting of 45 cycles of denaturation at 95°C for 10 sec and annealing/elongation at 60°C for 30 sec. We measured mRNA levels semi-quantitatively using the Δ/Δ threshold cycle (Ct) method. Both *glyceraldehyde-3-phosphate dehydrogenase* (*GAPDH*) and *phosphoglycerate kinase 1* (*PGK1*) were used simultaneously as reference genes. The values are expressed as relative to the SK-HEP-1 cells. Triplicate wells were analyzed in each assay.

### Flow cytometry

After cell cultivation, cells were dissociated with Accumax (Innovative Cell Technologies, San Diego, CA). Dissociated cells were then stained with Fixable Viability Dye eFluor 450 (eBioscience, San Diego, CA) to distinguish between living and dead cells. For flow cytometric analysis, the cells were incubated with the following fluorescence-conjugated antibodies: anti-CD44 APC (eBioscience), anti-CD24 APC (eBioscience), or anti-CD90 FITC (Miltenyi Biotec, Bergisch Gladbach, Germany). Rat IgG2b, k isotype control APC (eBioscience), mouse IgG1 APC isotype control (R&D Systems), and mouse IgG1 FITC isotype control (R&D Systems) were used as negative controls, respectively. For CD44 variant staining, anti-CD44v9 (Cosmo Bio, Tokyo, Japan) and mouse anti-rat IgG FITC (eBioscience) were used as primary and secondary antibodies, respectively. Rat IgG2a, k Isotype Control (eBioscience) was used as a negative control for the anti-CD44v9 antibody. Flow cytometric analysis was performed using a MACSQuant analyzer (Miltenyi Biotec).

Cell cycle distribution was analyzed with propidium iodide staining followed by flow cytometry. Cells were fixed with 70% ethanol and then resuspended in PI/RNase Staining Buffer (BD Biosciences, Franklin Lakes, NJ). The DNA content of cells was analyzed using a MACSQuant analyzer.

### Cell viability assay

The CellTiter 96 AQueous One Solution Cell Proliferation Assay (Promega, Tokyo, Japan), which includes 3-(4,5-dimethylthiazol-2-yl)-5-(3-carboxymethoxyphenyl)-2-(4-sulfophenyl)-2H-tetrazolium, inner salt (MTS) was used according to the manufacturer's instructions for evaluation of cell viability. Briefly, 5 × 10^3^ cells/well were seeded into 96-well plates and cultivated in sphere induction medium for 7 days to induce sphere cells. Then, an equal volume of the same medium or medium containing 5-fluorouracil (5-FU, Sigma-Aldrich), cisplatin (Sigma-Aldrich), carboplatin (Sigma-Aldrich), doxorubicin (Sigma-Aldrich), docetaxel (Sigma-Aldrich), suberoylanilide hydroxamic acid (SAHA, Cosmo Bio), irinotecan hydrochloride (Sigma-Aldrich), sunitinib malate (Sigma-Aldrich), or sorafenib tosylate (MBL, Nagoya, Japan) was added to the wells and incubated for 24-hours at 37°C in 5% CO_2_ in air. After incubation in anti-cancer drugs, MTS was added to the cells, which were then incubated for 2 additional hours at 37°C. The optical density of the culture medium at 492 and 650 nm was measured by using an EnVision plate reader (PerkinElmer, Waltham, MA). Triplicate wells were analyzed in each assay.

### Measurement of reactive oxygen species (ROS)

Intracellular ROS generation was measured with an OxiSelect ROS Assay Kit (CELL BIOLABS, San Diego, CA) according to the manufacturer's instructions. The cell membrane-permeable fluorescent dye 2’,7’-dichlorofluorescein diacetate (DCFH-DA) was added to cells. DCFH-DA is converted to the impermeable nonfluorescent compound DCFH by intracellular esterases. Highly fluorescent DCF is produced by oxidation of DCFH by ROS. The fluorescence intensity of DCF inside the cells was measured using an EnVision plate reader. Triplicate wells were analyzed in each assay.

### Statistical analysis

Each experiment was repeated at least three times, and data are expressed as the mean ± standard deviations. Data were compared using the Mann-Whitney *U*-test or repeated-measures analysis of covariance (ANCOVA), using SPSS Statistics 17.0 software (IBM, Tokyo, Japan). A *P* value of <0.05 was considered statistically significant.

## Results

### Induction of sphere cells from HCC cell lines

**Figure 1:**
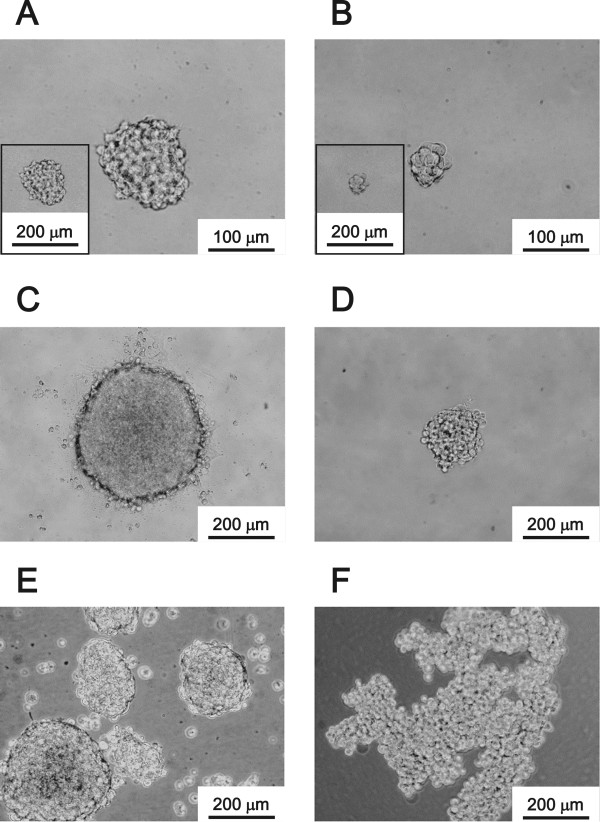
**Induction of sphere cells from HCC cell lines.** The sphere cells from a single cell at day 7 (***A***; SK-HEP-1, ***B***; HLE) and day 14 (***C***; SK-HEP-1, ***D***; HLE) in sphere induction culture medium. Induction began with a cell density of 1 × 10^5^ cells/mL, and cultivated for 7 days (***E***, from SK-HEP-1 and ***F***, from HLE).

**Figure 2:**
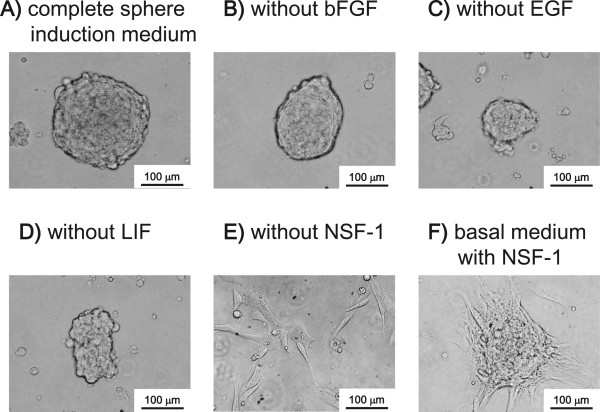
**Factors for sphere induction.** Sphere induction was performed with a cell density of 1 × 10^5^ SK-HEP-1 cells/mL for 7 days. ***A***, complete sphere induction medium was used. ***B***, ***C***, ***D***, and ***E***, complete induction medium lacking bFGF, EGF, LIF, or NSF-1, respectively, was used. ***F***, the basal medium was supplemented with only NSF-1.

Four human HCC cell lines, SK, HLE, Hep 3B and HuH-7, were used for induction of sphere cells. SK and HLE cells could form sphere cells (SK-spheres and HLE-spheres) from single cells (Figure 1). SK-spheres formed larger spheroids from single cells than HLE-spheres (Figure 1A-D). Sphere formation was more efficient, when starting with a high density of cells: 1 × 10^5^ cells/mL (Figure 1E and F). In the high-density condition, HLE cells formed floating spheroids and some adherent cells (Additional file 2: Figure S1A and B). Therefore, the floating cells were transferred into a hydrophilic culture flask on the next day of the sphere induction. In the hydrophilic flask, SK-spheres formed (Figure 1E), but HLE-spheres formed aggregated spheroids at day 7 (Figure 1F). In addition, SK-sphere and HLE-sphere cells could form spheroids again after dissociation (Additional file 2: Figure S1C and D). When the dissociated SK-sphere and HLE-sphere cells were returned to normal medium containing FBS, adherent cells formed again (Additional file 2: Figure S1E and F). Hep 3B and HuH-7 cells formed neither spheroids nor floating cells in these same conditions (Additional file 2: Figure S1G and H). Furthermore, in our sphere induction medium, no sphere cells were induced from SK cells when NSF-1 was not added (Figure 2). Conversely, basal medium supplemented with only NSF-1 could induce sphere cells from SK, although the spheroids obtained were adherent. Hence, de-differentiated HCC-derived cell lines, especially the SK cells, could form floating spheroids in our condition supplemented with NSF-1, but well-differentiated HCC-derived cell lines did not. According to these observations, we focused on the SK-sphere cells obtained after 7 days of induction from a high-density cell culture for the following analysis.

### Expression of stemness markers

**Figure 3:**
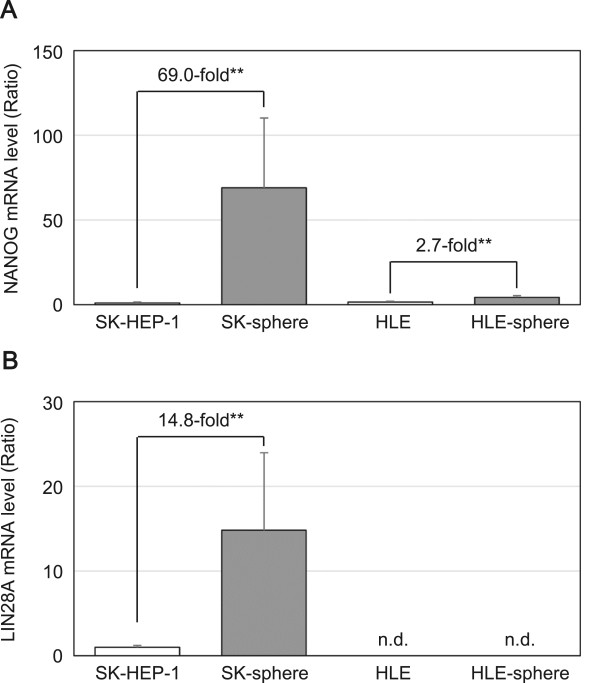
**mRNA levels of stemness markers in the induced sphere cells.** The mRNA levels of *NANOG* **(** ***A*** **)** and *LIN28A* **(** ***B*** **)** were measured with semi-quantitative RT-PCR and represented as the ratio to levels in SK-HEP-1 cells. **P < 0.05 with the Mann-Whitney *U*-test. n.d., not detectable in our experimental condition.

Our semi-qRT-PCR analysis showed that levels of *NANOG* and *LIN28A* mRNA in SK-sphere cells were higher than those in parental SK cells (Figure 3). Furthermore, the SK-sphere cells showed approximately 3-fold higher ALDH activity compared to SK cells (Additional file 3: Figure S2). However, sphere cells derived from HLE cells showed weak upregulation of the *NANOG* mRNA expression compared to the case of SK and SK-sphere cells (Figure 3A).

### Expression of CSC markers

**Figure 4:**
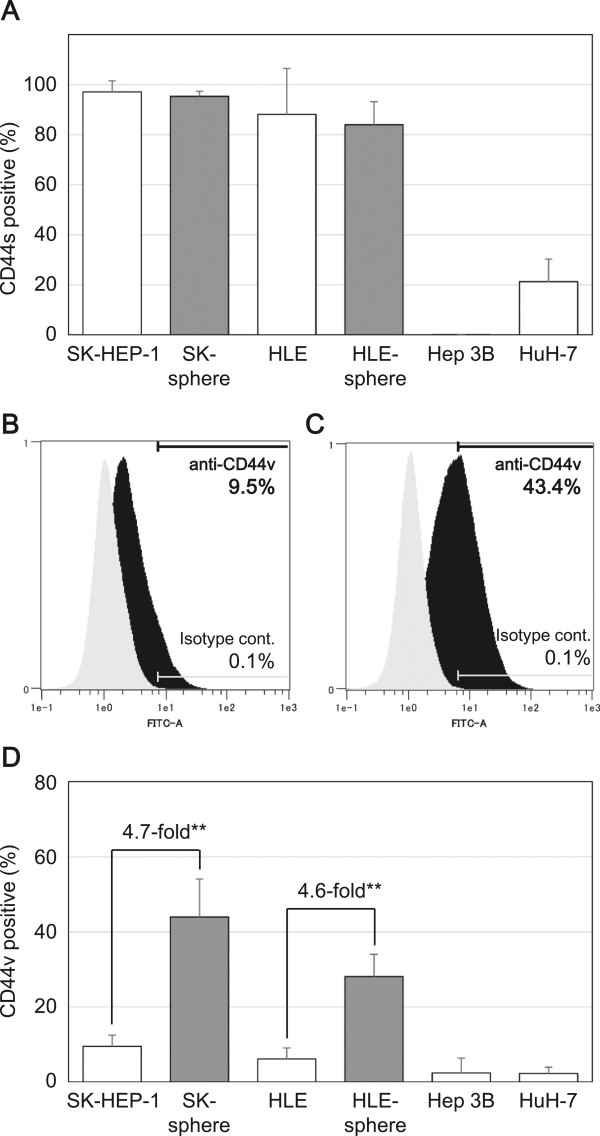
**Flow cytometric analysis of CD44 expression.** Cells were stained with APC-conjugated anti-CD44 antibody **(** ***A*** **)** or un-labeled anti-CD44 variant antibody followed by FITC-conjugated mouse anti-rat IgG antibody **(** ***B*** **to** ***D*** **)** and then separated with a flow cytometer. The positive for CD44s population in cells with sphere-forming potential, SK-sphere and HLE-sphere, were higher than those in non-sphere-forming cells **(** ***A*** **)**. ***B*** and ***C***, representative flow cytometry histograms of SK-HEP-1 and SK-sphere cells, respectively. Black and gray histograms represent cells stained with anti-CD44v and isotype control antibodies, respectively. ***D***, bar-chart summarized from independent flow cytometric experiments. Sphere cells, SK-sphere and HLE-sphere, showed an increased population of CD44v positive cells compared to their parental cells **(** ***D*** **)**. **P < 0.05 with the Mann-Whitney *U*-test.

**Figure 5:**
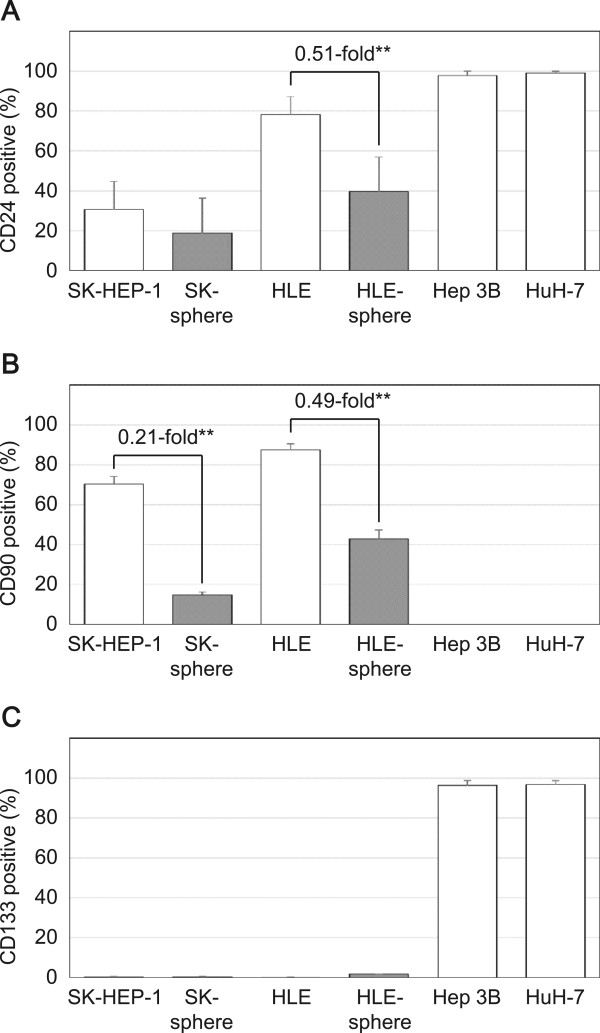
**Flow cytometric analysis of CD24, CD90, and CD133 expressions.** Cells were stained with APC-conjugated anti-CD24 antibody **(** ***A*** **)**, FITC-conjugated anti-CD90 antibody **(** ***B*** **)**, and PE-conjugated anti-CD133 antibody **(** ***C*** **)** and then separated with a flow cytometer. Sphere cells showed a decreased population of CD24 and CD90 positive cells than their parental cells. SK-HEP-1, HLE, and these derivative cells were negative for CD133.

We examined the expression levels of known CSC markers using flow cytometric analysis. The proportions of positive cells for CD44 standard isoform (CD44s) were higher in the sphere-forming SK and HLE cells than in the non-sphere-forming Hep 3B and HuH-7 cells (Figure 4A). Moreover, the proportions of positive cells for CD44 variant isoform (CD44v) were more than 4.5-fold higher in the sphere-formed SK-sphere and HLE-sphere cells than parental cells (*P* < 0.05, Figure 4B-D). In contrast, CD24- and CD90-positive cells were decreased in sphere-formed cells compared to parental cells (Figure 5A and B). Regarding CD133, nearly all SK and HLE cells were negative for anti-CD133 antibody, and we observed no induction and slight induction of CD133 expression in SK-sphere and HLE-sphere cells, respectively (Figure 5C).

### Susceptibility of SK-derived sphere cells to anti-cancer drugs

**Figure 6:**
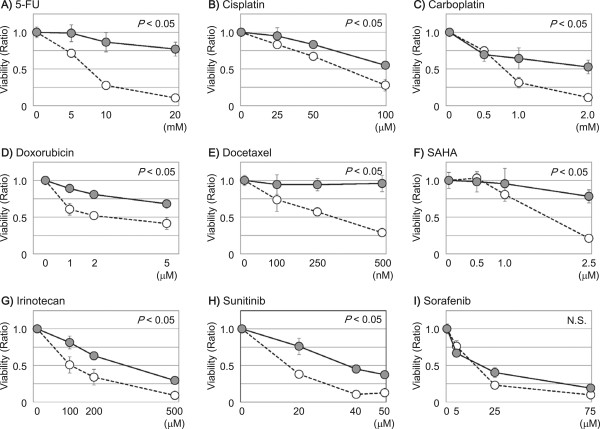
**Susceptibility of SK-HEP-1 derivative cells to anti-cancer drugs.** Cells were subjected to an MTS assay to evaluate the viability of SK-HEP-1 (open circles) and SK-sphere (closed circles) cells in the presence of anti-cancer drugs (***A***, 5-FU; ***B***, cisplatin; ***C***, carboplatin, ***D***, doxorubicin; ***E***, docetaxel; ***F***, SAHA; ***G***, irinotecan; ***H***, sunitinib; and ***I***, sorafenib). Except for sorafenib, SK-sphere cells showed higher viability in the presence of the tested anti-cancer drugs. *P* values were calculated with repeated-measures ANCOVA.

**Figure 7:**
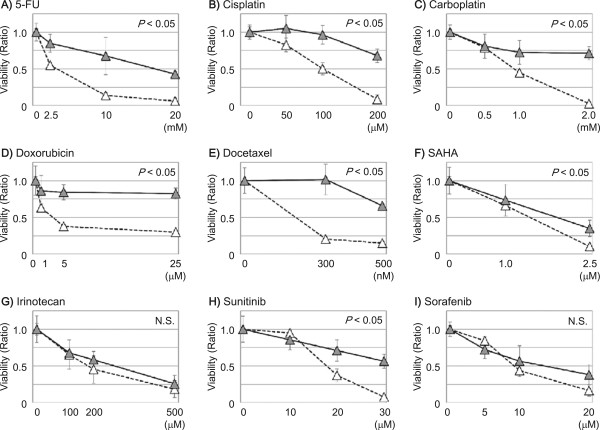
**Susceptibility of HLE derivative cells to anti-cancer drugs.** Cells were subjected to an MTS assay to evaluate the viability of HLE (open triangles) and HLE-sphere (closed triangles) cells in the presence of anti-cancer drugs (***A***, 5-FU; ***B***, cisplatin; ***C***, carboplatin, ***D***, doxorubicin; ***E***, docetaxel; ***F***, SAHA; ***G***, irinotecan; ***H***, sunitinib; and ***I***, sorafenib). *P* values were calculated with repeated-measures ANCOVA.

The susceptibility of SK and SK-sphere cells to several anti-cancer drugs was evaluated by measuring the viability of cells treated with anti-cancer drugs for 24 hours (Figure 6). SK-sphere cells showed significantly higher viability in medium containing 5-FU, cisplatin, carboplatin, doxorubicin, docetaxel, SAHA, irinotecan, and sunitinib compared to SK cells (1.7 to 7.3-fold at each of the highest doses, *P* < 0.05). Interestingly, no significant anti-cancer effect was seen with sorafenib between SK-sphere cells and SK cells (Figure 6I). Similarly, HLE-sphere cells showed increased chemoresistace compared to HLE cells (Figure 7). As a result, both of obtained sphere cells showed tolerance to several types of anti-tumor drugs.

### Expression of ATP-binding cassette (ABC) transporters

**Figure 8:**
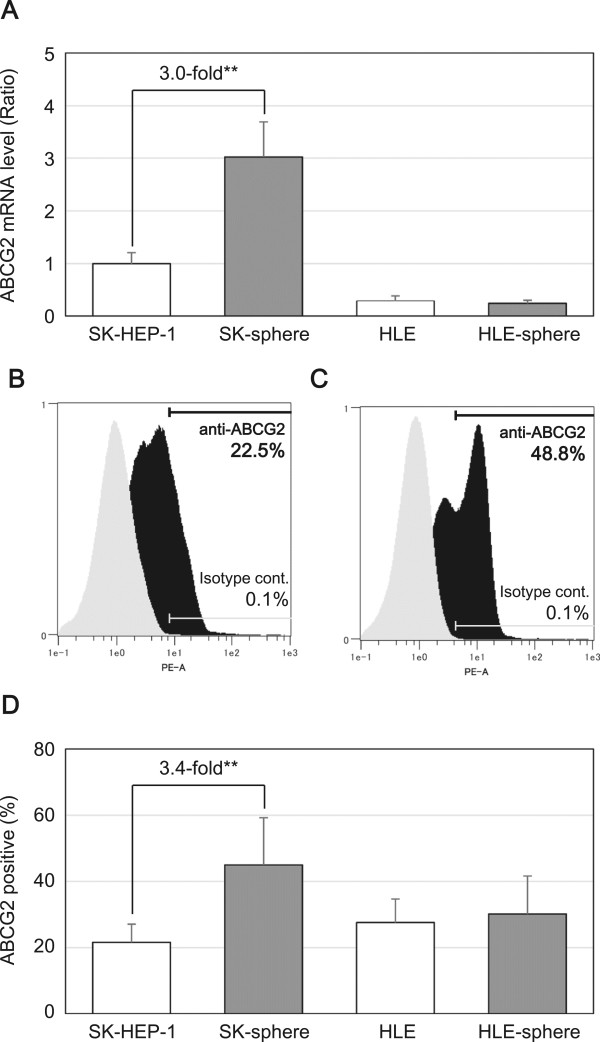
**Expression levels of ABCG2.** The expression levels of ABCG2 mRNA **(** ***A*** **)** and protein **(** ***B*** **to** ***D*** **)** were examined with semi-quantitative RT-PCR and flow cytometry, respectively. ***B*** and ***C***, representative flow cytometry histograms of SK-HEP-1 and SK-sphere cells, respectively. Black and gray histograms represent cells stained with PE-conjugated anti-ABCG2 and isotype control antibodies, respectively. ***D***, bar-chart summarized from independent flow cytometric experiments. **P < 0.05 with the Mann-Whitney *U*-test.

Using semi-qRT-PCR and flow cytometry, we measured the mRNA and protein expression levels, respectively, of ABCG2, which is an ABC transporter. SK-sphere cells showed approximately 3.0-fold higher ABCG2 mRNA levels compared to SK cells (*P* < 0.05, Figure 8A). Similarly, protein expression levels of ABCG2 were higher in SK-sphere cells than parental cells (Figure 8B-D). On the other hand, HLE and HLE-sphere cells showed no significant difference in the ABCG2 expression (Figure 8D).

### Cell cycle distribution in sphere forming cells

**Figure 9:**
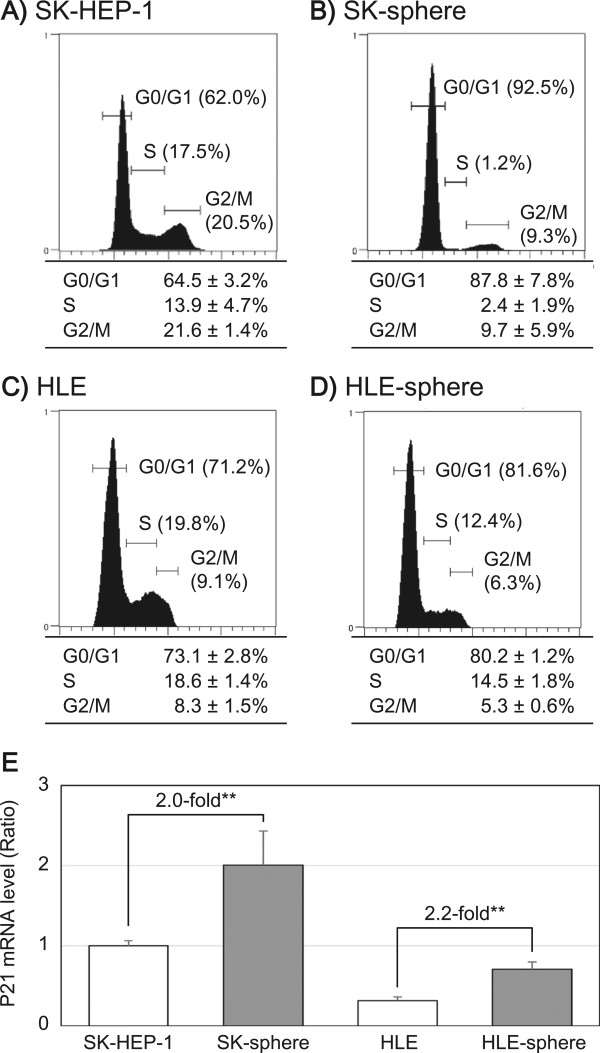
**Cell cycle analysis.** Cell cycle distribution of SK-HEP-1 **(** ***A*** **)**, SK-sphere **(** ***B*** **)**, HLE **(** ***C*** **)**, and HLE-sphere **(** ***D*** **)** cells, respectively. DNA content of the cells was analyzed as described in Methods. Each panel **(** ***A*** **to** ***D*** **)** shows a representative histogram (upper) and a summary table (lower). ***E***, The mRNA levels of *P21* were measured with semi-quantitative RT-PCR. **P < 0.05 with the Mann-Whitney *U*-test.

SK cells showed a cell cycle distribution of 64.5 ± 3.2% at G0/G1, 13.9 ± 4.7% at S, and 21.6 ± 1.5% at G2/M phases. In contrast, SK-sphere cells showed a cell cycle distribution of 87.8 ± 7.8% at G0/G1, 2.5 ± 1.9% at S, and 9.7 ± 5.9% at G2/M phases (Figure 9A and B). HLE-sphere cells also showed increased proportion of G0/G1 phase compared to HLE cells (Figure 9C and D). Furthermore, mRNA expression levels of *P21*, which encode the cyclin-dependent kinase inhibitor 1, were increased in SK-sphere (2.0-fold, *P* < 0.05) and HLE-sphere (2.2-fold, *P* < 0.05) cells compared to parental cells (Figure 9E).

### HIF1α expression and ROS levels

**Figure 10:**
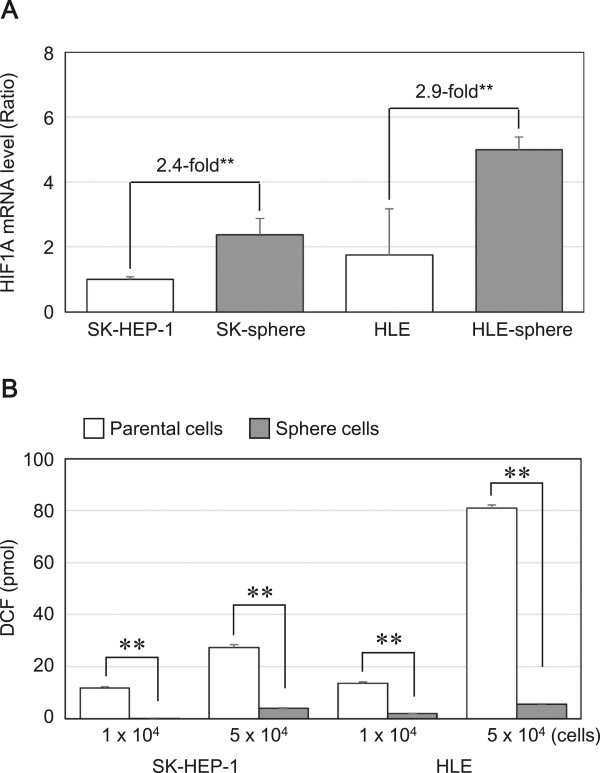
***HIF1A*** **mRNA and ROS levels.** The mRNA level of *HIF1A* **(** ***A*** **)** and the intracellular ROS level **(** ***B*** **)** were measured as described in Methods. **P < 0.05 with the Mann-Whitney *U*-test.

We examined mRNA levels of *HIF1A* encoding hypoxia inducible factor 1, alpha subunit (HIF1α) and intracellular ROS levels using semi-qRT-PCR analysis and a fluorogenic probe that is activated by ROS, respectively. Semi-qRT-PCR showed that the mRNA levels of *HIF1A* were approximately 2.4-fold higher in SK-sphere cells than parental cells (*P* < 0.05, Figure 10A). Furthermore, lower ROS activity was observed in SK-sphere cells than in parental cells (approximately 0.1-fold, *P* < 0.05, Figure 10B). HLE cells also showed that the higher *HIF1A* expression and lower ROS production in sphere-formed cells than its parental cells (Figure 10). Those results indicated that sphere cells from both SK and HLE cells had elevated antioxidant potential.

## Discussion

In the present study, we developed a novel method using NSF-1-supplemented medium to induce sphere cells from an SK cell line, which was derived from a human poorly differentiated HCC. HLE cells, which were derived from an undifferentiated HCC, also formed sphere-like cells. On the other hand, using the same culture method, no sphere cells were derived from Hep 3B and HuH-7 cells, which are both derived from well-differentiated HCC. The SK-sphere cells showed higher expression levels of CSC markers and higher anti-cancer drug resistance compared to parental SK cells. Furthermore, we also observed up-regulation of ABCG2 expression, cell cycle arrest at G0/G1, overexpression of HIF1α, and decreased ROS production in SK-sphere cells compared to parental SK cells.

Recently, sphere formation has been thought to be an effective method of enrichment or induction of stem cell-like cells from various types of cancers [16, 17, 27]. The advantage of induction and enrichment of CSCs is that cell sorting, which damages cells, is not required. Usually, sphere formation is done with serum-free medium supplemented with bFGF and EGF [15, 17, 28, 29]. In addition to bFGF and EGF, we added NSF-1, which is a key factor for SK-sphere induction (Figure 2). In our condition, numerous cancer stem-like sphere cells were obtained easily. Several studies have used B27 and N2 supplements instead of NSF-1 to form sphere cells [15–17, 27, 29]. NSF-1-containing medium was more efficient than B27-supplemented medium for inducing sphere cells (data not shown). Furthermore, our induction system could generate a sphere from a single cell (Figure 1).

Interestingly, in our condition, sphere cells were induced from the poorly or un-differentiated HCC-derived cell lines, SK and HLE, but not from the well-differentiated HCC-derived cell lines, Hep 3B and HuH-7. The SK-spheres we obtained showed increased expression of stemness markers and CD44 isoform expression (Figures 3 and 4). *NANOG* and *LIN28*, which are induced pluripotent stem cell-related genes and master transcription factors essential for maintaining stem cell phenotypes [30, 31], and high ALDH activity have been found in stem cell populations in various types of cancers [32, 33]. CD44 variant isoforms have been identified as markers for CSCs and metastatic behavior in several solid cancers [7, 34]. On the contrary, CD24 expression was down-regulated in sphere cells (Figure 5). Down-regulation of CD24 was observed in CSCs of breast cancer [35]. However, a well-known CSC marker for HCC, CD133 [8, 9, 13], was not expressed in SK- and HLE-spheres (Figure 5). Moreover, the strongly CD133-positive cell lines, Hep 3B and HuH-7, did not form spheres. Previous studies have shown that well-differentiated hepatoma-derived cell lines contain more CSCs than poorly differentiated hepatoma-derived cell lines [22]. These findings suggest the existence of a difference between CSCs at the top of the hierarchy model that are responsible for primary cancer initiation [18] and CSCs derived from progressed cancer with plasticity [23]. Such contextual signals within the cancer microenvironment and EMT contribute to the plasticity of cancer, which involves transformation to cancer stem-like cells [36]. Indeed, expression of the mesenchymal marker vimentin was up-regulated in SK-sphere cells (data not shown).

Many studies have indicated that CSCs play a critical role in chemoresistance [9, 37, 38]. Our induced cancer stem-like sphere cells, SK-spheres and HLE-spheres, showed lower susceptibility to 5-FU, cisplatin, carboplatin, doxorubicin, docetaxel, SAHA, and sunitinib than their parental cells. One well-known mechanism of chemoresistance is up-regulation of ABC transporters, which mediate efflux of the anti-cancer drugs [37–39]; expression of one ABC transporter, ABCG2, was up-regulated in SK-sphere cells (Figure 8). However, HLE-sphere and HLE cells showed no significant difference about ABCG2 expression (Figure 8). CSC dormancy is also responsible for chemoresistance of CSCs [40]. SK-sphere cells were arrested at the G0/G1 phase of the cell cycle compared to parental SK cells, and the *P21* mRNA level was higher in SK-sphere cells than in SK cells (Figure 9). P21 plays an important role in the transition of the cell cycle from the G0/G1 to the S phase [41, 42].

ROS are also important factors for chemoresistance of cancer. In SK-sphere and HLE-sphere cells, up-regulation of HIF1α expression and decreased levels of ROS were observed (Figure 10). CSCs contained lower ROS levels and developed less DNA damage compared with non-CSCs. HIF-1α, which is a member of the major family of transcription factors that are activated by hypoxia, is an important contributor to reduction of ROS production [28, 43, 44]. Furthermore, SK-sphere and HLE-sphere cells had more cells that were positive for the CD44 variant isoform (Figure 4), which stabilizes the xCT transporter and enhance antioxidant synthesis [45].

Among the anti-cancer drugs we tested, only sorafenib exerted an anti-cancer effect on both sphere cells and parental cells (Figures 6 and 7). Although both sorafenib and sunitinib are multi-targeted receptor tyrosine kinase inhibitor, sorafenib inhibits RAS/RAF/MAPK signaling, and treatment of patients with advanced HCC with sorafenib provides longer overall survival than sunitinib [46]. Therefore, sorafenib may be useful against both HCC and CSCs from HCC.

Although HLE-sphere cells as well as SK-sphere cells showed significantly increased resistance to several anti-cancer drugs and antioxidant potentials compared to parental cells, sphere cells from HLE cells seem to be cells bearing cancer stem-like properties with incompleteness in morphology, stemness marker, and ABCG2 expressions.

## Conclusion

In conclusion, we developed a novel method for induction of cancer stem-like chemoresistant sphere cells from cell lines derived from de-differentiated HCC involving short-term cultivation.

## Electronic supplementary material

Additional file 1: Table S1: Primers and hydrolysis probes used in this study. (XLS 8 KB)

Additional file 2: Figure S1: Sphere induction from HCC cell lines. A and B, HLE cells cultivated in the sphere induction medium for 4 days. There was floating sphere cells and adherent cells. Dissociated SK-sphere and HLE-sphere cells were re-cultivated in the induction medium for 7 days (C and D, photographs of passage 3) and normal medium containing FBS (E and F). Hep3B (G) and HuH-7 (H) cells in the same sphere induction medium, but there was no spheroids nor floating cells. (PDF 381 KB)

Additional file 3: Figure S2: ALDH expression and activity. The mRNA levels of *ALDH1A1* were measured with semi-quantitative RT-PCR and represented as the ratio to levels in SK-HEP-1 cells (A). **P< 0.05 with the Mann-Whitney *U*-test. The ALDEFLUOR kit (STEMCELL Technologies, Durham, NC) was used to analyze the ALDH enzymatic activity in a population of cells. Band C, SK-HEP-1 and SK-sphere cells were suspended in ALDEFLUOR assay buffer containing ALDH substrate and incubated for 40 min at 37°C. D and E, as a negative control, an aliquot of SK-HEP-1 and SK-sphere cells was treated with 50 mM diethylaminobenzaldehyde (DEAB), a specific ALDH inhibitor. (PDF 324 KB)
