# Supplementary material for: Cancer stem-like sphere cells induced from de-differentiated hepatocellular carcinoma-derived cell lines possess the resistance to anti-cancer drugs
Source: BMC Cancer. 2014 Sep 27;14:722. doi: 10.1186/1471-2407-14-722 (PMC4190290; doi:10.1186/1471-2407-14-722)
Supplement: Supplementary file 2 — Additional file 2: Figure S1: Sphere induction from HCC cell lines. A and B, HLE cells cultivated in the sphere induction medium for 4 days. There was floating sphere cells and adherent cells. Dissociated SK-sphere and HLE-sphere cells were re-cultivated in the induction medium for 7 days (C and D, photographs of passage 3) and normal medium containing FBS (E and F). Hep3B (G) and HuH-7 (H) cells in the same sphere induction medium, but there was no spheroids nor floating cells. (PDF 381 KB) [file 12885_2014_4908_MOESM2_ESM.pdf]

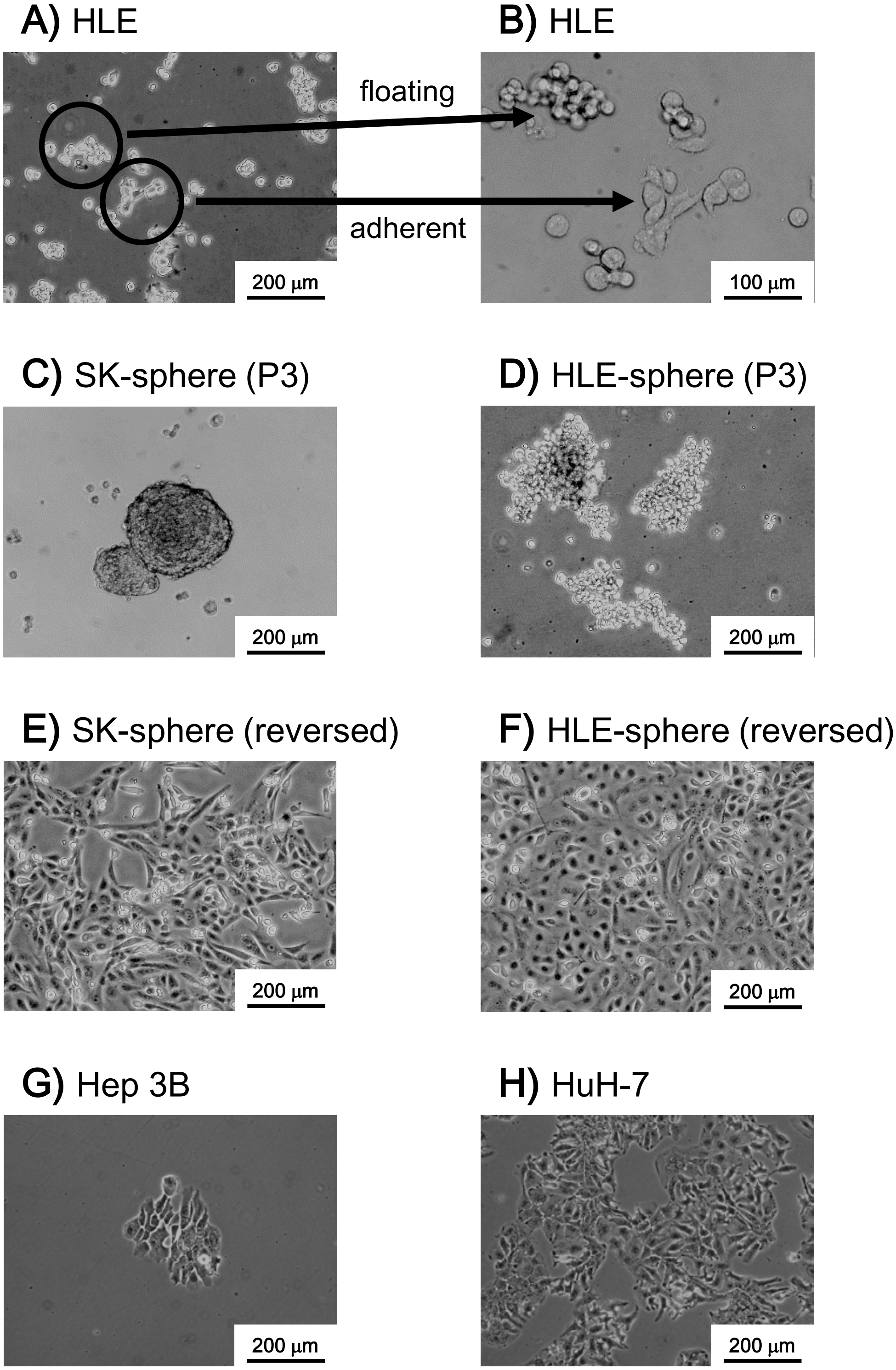

Supplemental Figure S1 Sphere induction from HCC cell lines. *A* and *B*, HLE cells cultivated in the sphere induction medium for 4 days. There was floating sphere cells and adherent cells. Dissociated SK-sphere and HLE-sphere cells were re-cultivated in the induction medium for 7 days (*C* and *D*, photographs of passage 3) and normal medium containing FBS (*E* and *F*). Hep 3B (*G*) and HuH-7 (*H*) cells in the same sphere induction medium, but there was no spheroids nor floating cells.
