# Supplementary material for: Cancer stem-like sphere cells induced from de-differentiated hepatocellular carcinoma-derived cell lines possess the resistance to anti-cancer drugs
Source: BMC Cancer. 2014 Sep 27;14:722. doi: 10.1186/1471-2407-14-722 (PMC4190290; doi:10.1186/1471-2407-14-722)
Supplement: Supplementary file 3 — Additional file 3: Figure S2: ALDH expression and activity. The mRNA levels of ALDH1A1 were measured with semi-quantitative RT-PCR and represented as the ratio to levels in SK-HEP-1 cells (A). **P< 0.05 with the Mann-Whitney U-test. The ALDEFLUOR kit (STEMCELL Technologies, Durham, NC) was used to analyze the ALDH enzymatic activity in a population of cells. Band C, SK-HEP-1 and SK-sphere cells were suspended in ALDEFLUOR assay buffer containing ALDH substrate and incubated for 40 min at 37°C. D and E, as a negative control, an aliquot of SK-HEP-1 and SK-sphere cells was treated with 50 mM diethylaminobenzaldehyde (DEAB), a specific ALDH inhibitor. (PDF 324 KB) [file 12885_2014_4908_MOESM3_ESM.pdf]

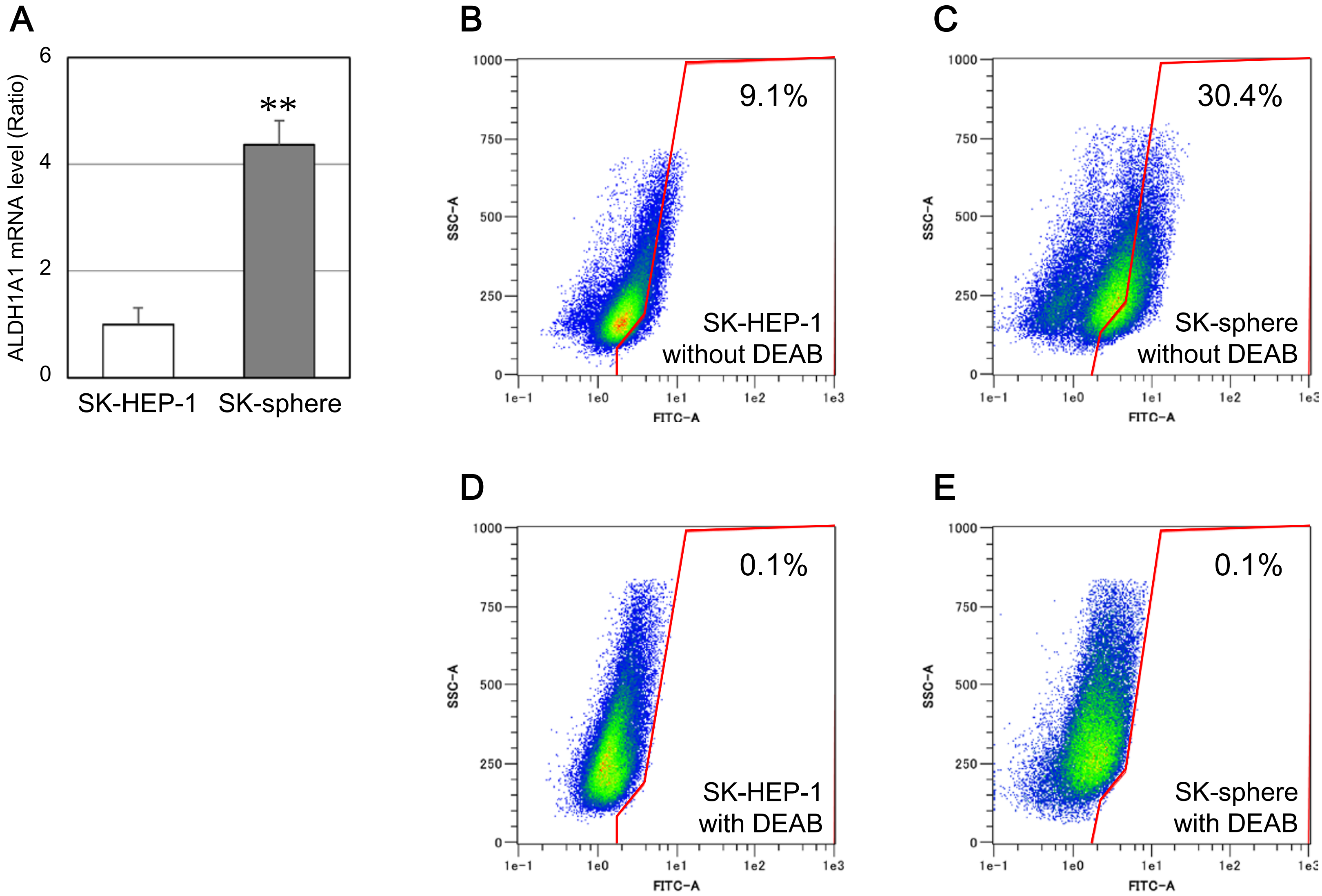

Supplemental Figure S2 ALDH expression and activity. The mRNA levels of ALDH1A1 were measured with semi-quantitative RT-PCR and represented as the ratio to levels in SK-HEP-1 cells (*A*). ***P* < 0.05 with the Mann-Whitney *U*-test. The ALDEFLUOR kit (StemCell Technologies, Durham, NC) was used to analyze the ALDH enzymatic activity in a population of cells. *B* and *C*, SK-HEP-1 and SK-sphere cells were suspended in ALDEFLUOR assay buffer containing ALDH substrate and incubated for 40 min at 37 °C. *D* and *E*, as a negative control, an aliquot of SK-HEP-1 and SK-sphere cells was treated with 50 mM diethylaminobenzaldehyde (DEAB), a specific ALDH inhibitor.
